# Acute pancreatitis with saw palmetto use: a case report

**DOI:** 10.1186/1752-1947-5-414

**Published:** 2011-08-25

**Authors:** Jackrapong Bruminhent, Perliveh Carrera, Zhongzhen Li, Raymond Amankona, Ingram M Roberts

**Affiliations:** 1 University of Connecticut School of Medicine, Department of Internal Medicine, St Vincent's Medical Center, 2800 Main Street, Bridgeport, Connecticut, 06606, USA

## Abstract

**Introduction:**

Saw palmetto is a phytotherapeutic agent commercially marketed for the treatment of benign prostatic hyperplasia. Evidence suggests that saw palmetto is a safe product, and mild gastrointestinal adverse effects have been reported with its use. We report a case of acute pancreatitis, possibly secondary to the use of saw palmetto.

**Case presentation:**

A 61-year-old Caucasian man with a history of benign prostatic hyperplasia and gastroesophageal reflux disease developed epigastric pain associated with nausea 36 hours prior to presentation. He denied drinking alcohol prior to the development of his symptoms. His home medications included saw palmetto, lansoprazole and multivitamins. Laboratory results revealed elevated lipase and amylase levels. An abdominal ultrasound demonstrated a nondilated common bile duct, without choledocholithiasis. Computed tomography of his abdomen showed the pancreatic tail with peripancreatic inflammatory changes, consistent with acute pancreatitis. Our patient's condition improved with intravenous fluids and pain management. On the fourth day of hospitalization his pancreatic enzymes were within normal limits: he was discharged home and advised to avoid taking saw palmetto.

**Conclusion:**

It is our opinion that a relationship between saw palmetto and the onset of acute pancreatitis is plausible, and prescribers and users of saw palmetto should be alert to the possibility of such adverse reactions.

## Introduction

*Serenoa repens *(W. Bartram) Small, more commonly known as saw palmetto or scrub palmetto, is a low-growing palm endemic to the Southeastern United States [1]. Ecologically, saw palmetto is used for nesting, protective cover and as a food source by wildlife. The medicinal value of the fruit among humans has been described in scientific literature since the 1800s for the relief of prostate gland swelling [2].

The liposterolic extract of saw palmetto has antiandrogenic activity in human prostatic cell lines [3]. Furthermore, it inhibits binding of dihydrotestosterone (DHT) to its receptor [4] and prevents the conversion of testosterone into DHT by inhibiting the activity of 5-alpha-reductase [5], exhibiting a similar mechanism to the Food and Drug Administration (FDA) -approved medication finasteride. *In vitro *studies have shown that it also inhibits cyclooxygenase and 5-lipoxygenase pathways, thereby preventing the biosynthesis of inflammation-producing prostaglandins and leukotrienes [6].

As a phytotherapeutic agent, saw palmetto is currently being commercially marketed for the treatment of benign prostatic hyperplasia (BPH). A systematic review of randomized control trials of saw palmetto, involving 2939 men, reported similar improvement in the urologic symptoms of BPH and urinary flow measures compared with finasteride. Furthermore, it was associated with fewer adverse effects [7]. However, a more recent double-blind randomized control trial of 225 men, comparing a saw palmetto and a placebo group, showed no significant difference in their American Urological Association Symptom Index scores or maximal urinary flow rate, with the incidence of side effects similar between the two groups [8]. The latest Cochrane review with 5222 subjects from 30 randomized trials also confirmed that saw palmetto was well tolerated, but was no better than placebo in improving urinary symptoms, peak urine flow, or prostate size for men with BPH [9].

Amidst conflicting data on its utility as treatment for BPH, current evidence suggest that saw palmetto is well-tolerated by patients and is not associated with serious adverse events. Concomitantly, most adverse events reported were mild, infrequent and reversible. A systematic review of adverse events by Agbabiaka *et al*. from monopreparations of saw palmetto suggests that adverse events associated with its use are similar to those occurring in placebo groups. Adverse effects that have been frequently reported include abdominal pain, diarrhea, nausea and fatigue, headache, decreased libido and rhinitis [10].

More serious adverse effects have also been reported in isolated case reports, including protracted cholestatic hepatitis after the use of Prostata, an herbal preparation used for prostatic hypertrophy with saw palmetto as presumably the most active ingredient [11], and acute pancreatitis. To date there have been two case reports of acute pancreatitis associated with saw palmetto use [12,13]. Acute pancreatitis accounts for more than 200,000 hospital admissions annually in the United States and its hospitalization rate is rising [14]. It ranks third in the list of hospital discharges for gastrointestinal related diseases. Mortality from acute pancreatitis is less than 5% overall, but severe cases cause prolonged hospitalization and significantly higher mortality [15]. We report a case of acute pancreatitis, possibly secondary to the use of saw palmetto, and review the other cases in the literature of this condition.

## Case presentation

A 61-year-old Caucasian man with a history of BPH and gastroesophageal reflux disease, who has been in his usual state of health until 36 hours prior to presentation, developed epigastric pain characterized as dull, constant, non-radiating, aggravated by positional changes without any alleviating factors and associated with nausea. He denied any similar episode of abdominal pain in the past. On physical examination, our patient was febrile at 38.5°C and tachycardic; his abdomen was soft with epigastric and periumbilical tenderness and minimal guarding. He occasionally drank a bottle of beer every two to three weeks but denied drinking alcohol recently, had a remote smoking history, and denied any illicit drug use. His home medications included saw palmetto, which he had been taking for the past three years, lansoprazole and multivitamins. His BPH was initially treated with tamsulosin by his urologist, however he experienced dizziness with this medication and was unable to tolerate it. He was then prescribed saw palmetto, which offered relief for his BPH symptoms.

Laboratory results upon admission revealed elevated lipase and amylase levels at 4406 units/L (reference range, RR 20-104 units/L) and > 3500 units/L (RR 5.6-51.3 units/L), respectively. Triglycerides were normal at 145 mg/dL (RR < 250 mg/dL); his alcohol level was less than 10 mg/dL (RR 0-80 mg/dL).

Our patient's liver function tests were normal: aspartate transaminase 35 units/L (RR 8-20 units/L), alanine transaminase 33 units/L (RR 10-40 units/L), alkaline phosphatase 140 units/L (RR 27-100 units/L) and total bilirubin 0.6 mg/dL (RR < 20 mg/dL). Basic metabolic panels were also within normal limits. His calcium level was 9.3 mg/dL (RR 8.5 10.4 mg/dL).

**Figure 1:**
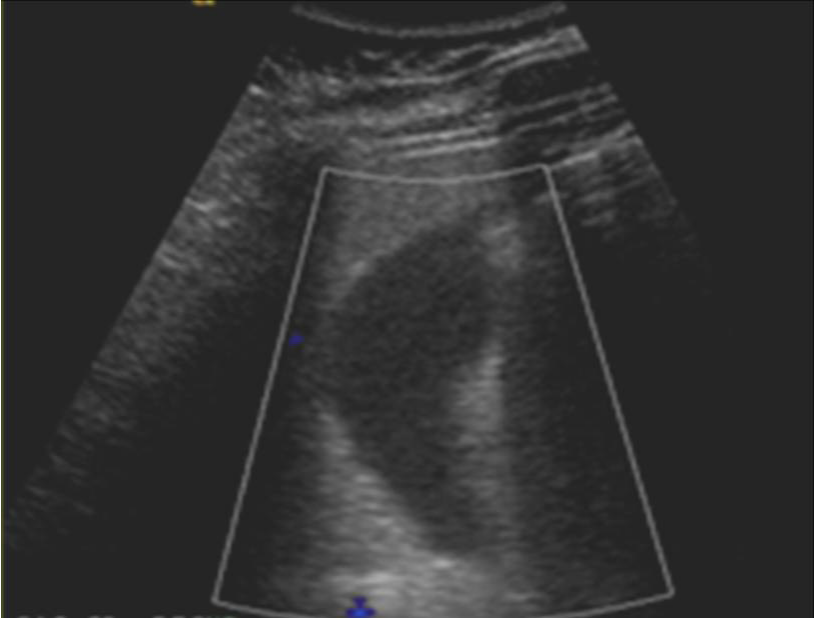
**Abdominal ultrasonography showed no evidence for gallstones; common bile duct was not dilated and measured 0.5 cm**.

**Figure 2:**
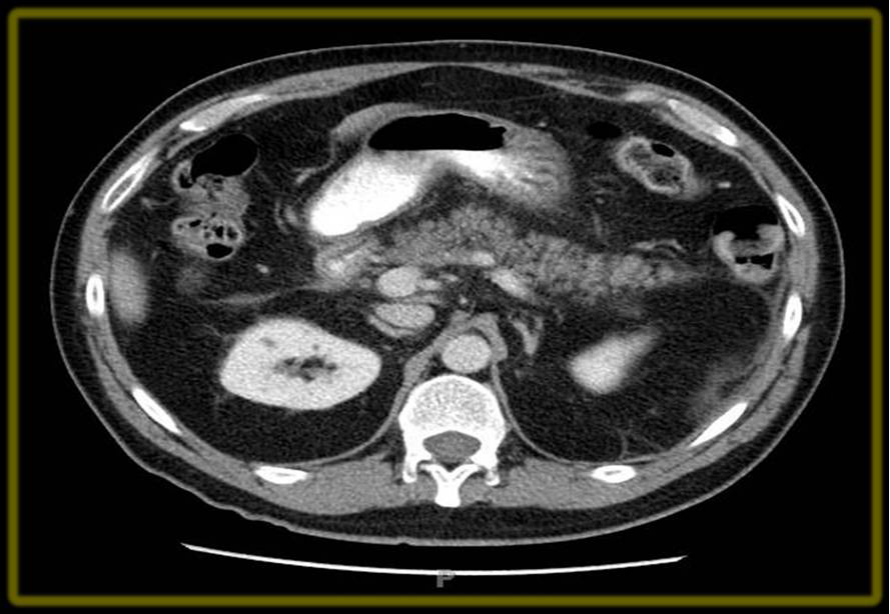
**Abdominal CT scan with contrast showed an indistinct pancreatic tail with peripancreatic inflammatory changes consistent with pancreatitis**.

A complete blood count was unremarkable except for leukocytosis at 14.1 × 10^3^cells/mm^3^. An abdominal ultrasound demonstrated a common bile duct, measuring 0.5 cm in diameter, without cholelithiasis (Figure 1). Computed tomography (CT) of his abdomen with contrast showed that his pancreatic tail was indistinct with peripancreatic inflammatory changes, consistent with acute pancreatitis (Figure 2). Our patient was diagnosed with acute pancreatitis and treated with supportive care, which included intravenous fluids and pain management. Our patient's pain improved, his diet was slowly advanced, and home medications were resumed with the exception of saw palmetto. On the fourth day of hospitalization, his pancreatic enzymes were within normal limits; he was discharged home with a lipase of 32 units/L and advised to avoid taking saw palmetto.

## Discussion

The most common causes for pancreatitis in adults are cholelithiasis and excessive alcohol use, accounting for 35-40% and 30% of cases, respectively. Other causes include anatomic variants of the pancreas, mechanical obstruction to pancreatic juice, hypertriglyceridemia, hypercalcemia, drug induced, toxins, trauma, ischemia, infections and autoimmune conditions [16]. Many medications also have been identified as a probable cause of acute pancreatitis. The first to report a case of drug-induced acute pancreatitis was Zion *et al*. in 1955; they described a case of hemorrhagic pancreatitis associated with cortisone therapy [17]. Drug-induced pancreatitis is rare, although more than 100 drugs have been implicated in causing this condition. It is rarely accompanied by clinical or laboratory evidence of a drug reaction, such as eosinophilia and rash [18]. Definite association of drugs with acute pancreatitis include aminosalicylates, L-asparaginase, azathioprine, didanosine, estrogen, furosemide, pentamidine, sulfonamide, tetracycline, thiazides, valproic acid, vinca alkaloids and 6-mercaptopurine [16].

A detailed history and physical examination along with routine radiological evaluation consisting of ultrasound and/or CT of the abdomen can detect the underlying etiology of acute pancreatitis in approximately 80% of patients. If this initial investigation is unrevealing, the patient is classified as having idiopathic acute pancreatitis [19].

In our patient, cholelithiasis, hypertriglyceridemia, infection and trauma were ruled out as possible causes of pancreatitis. In addition, as per the history of our patient, he had no recent alcohol use and consumption was very minimal.

Though confirmation of microlithiasis and anatomic variants of the pancreas such as papillary stenosis and sphincter of Oddi dysfunction are most accurately obtained using endoscopic ultrasonography, magnetic resonance cholangiopancreatography or sphincter of Oddi manometry [20], these procedures were not pursued in our patient because of his clinical presentation and subsequent improvement.

The required extensiveness of a search for the etiology in a patient with a first episode of unexplained pancreatitis is still a matter of debate [21]. A retrospective study which looked at patients presenting with a first episode of unexplained acute pancreatitis showed that only about 3.2% (one in 31 cases) would suffer another attack during a median follow-up of 36 months [22], suggesting that extensive investigation for unusual causes of pancreatitis may not required after the first episode of unexplained pancreatitis. Furthermore, invasive testing is associated with procedure-related complications and the relationship between some of those findings and the etiology of the pancreatitis is not always clear [23]. Based on local expertise, advanced evaluation is definitely indicated in patients with a severe initial attack of acute pancreatitis or with two or more attacks [19].

An association between acid suppressing drugs and acute pancreatitis has not been clearly supported by cohort and case control studies. Although proton pump inhibitors like omeprazole, pantoprazole and rabeprazole have been implicated in drug-induced pancreatitis, no case report for other proton pump inhibitors like lansoprazole has been described [24,25].

Apart from our patient and two other case reports, no other data on saw palmetto-induced acute pancreatitis have been reported. Our case and previous case reports (Table 1) reveal consistent resolution of symptoms and pancreatic enzymes following discontinuation during brief hospitalization (three to four days) which is consistent with the short half-life of saw palmetto [26]. To the best of our knowledge, this is the first reported case of acute pancreatitis with a normal alanine transaminase level, which goes against the probability of gallstone pancreatitis [27].

**Table 1:** Characteristics of three patients with saw palmetto-induced pancreatitis

Case Number [Ref#]	Age (years)	Sex	Amylase (Units/L)	Lipase (Units/L)	AST (Units/L)	ALT (Units/L)	Days of resolution
1 [12]	55	M	2152	39,346	1265	1232	four

2 [13]	65	M	626	2697	n/a	n/a	three

3 [our case]	61	M	4406	> 3500	35	33	four

ALT: alanine transminase; AST: aspartate transminase

In a recent review by Badalov *et al*. of reports on drug-induced acute pancreatitis from 1955 to 2006, they classified reported medications into four classes based on the published weight of evidence for each agent and the pattern of clinical presentation. Class I included medications in which at least one case was proven by a re-challenge with the drug. Class II included drugs with a consistent latency in 75% or more of the reported cases. Class III included drugs that had two or more case reports published, but neither a re-challenge nor a consistent latency period. Class IV drugs were similar to class III drugs, but only one case report had been found [18]. Based on the aforementioned classification, saw palmetto could be placed as a Class III agent for drug-induced acute pancreatitis.

The mechanisms of action for drug-induced acute pancreatitis are based on theories extracted from case reports, case-control studies, animal studies and other experimental data. In general, some potential mechanisms of action for drug-induced acute pancreatitis include pancreatic duct constriction, cytotoxic and metabolic effects, accumulation of a toxic metabolite or intermediary and hypersensitivity reactions [28]. A mechanism for saw palmetto-induced pancreatitis has not been thoroughly established. One theory suggests that it occurs through its estrogenic effects by stimulating estrogen receptors; it then induces a hypercoagulable state that leads to pancreatic necrosis [29]. However, as in the two previous case reports, pancreatic necrosis was not observed in our patient.

It is also important to note that the United States FDA regulate dietary supplements under a different set of regulations than those covering "conventional" foods and drug products, where the dietary supplement manufacturer is responsible for ensuring that a supplement is safe before it is marketed. FDA is responsible for taking action against any unsafe supplement product after it reaches the market; hence the lack of a standard premarketing regulation for dietary supplements [30].

## Conclusion

Current data show that the risk of drug-induced acute pancreatitis is low and it is imperative to rule out more common causes before attributing the event to a certain medication. Three case reports of probable saw palmetto-induced pancreatitis have been described and all the patients had been taking the medication for BPH symptoms. Even with a relatively safe profile as shown by studies on patients taking saw palmetto, the risk of having an adverse reaction exists and warrants immediate withdrawal of the drug and further investigation to prevent serious consequences.

Except for the fact that our patient took saw palmetto, there was no established cause of his acute pancreatitis. Our case highlights the importance of taking a detailed medication history including phytotherapeutic agents in all patients and prompt discontinuation of a probable offending drug to ensure patient safety.

## Abbreviations

BPH: benign prostatic hyperplasia; CT: computed tomography; DHT: dihydrotestosterone; FDA: Food and Drug Administration; RR: reference range.

## Consent

Written informed consent was obtained from the patient for publication of this case report and any accompanying images. A copy of the written consent is available for review by the Editor-in-Chief of this journal.

## Competing interests

The authors declare that they have no competing interests.

## Authors' contributions

JB, PC and RA contributed to the patient's clinical care. JB, PC and ZL drafted the manuscript. IR reviewed the manuscript. All authors revised and approved the final manuscript.
